# Supplementary material for: Heat-Inactivation of Fetal and Newborn Sera Did Not Impair the Expansion and Scaffold Engineering Potentials of Fibroblasts
Source: Bioengineering (Basel). 2021 Nov 13;8(11):184. doi: 10.3390/bioengineering8110184 (PMC8615100; doi:10.3390/bioengineering8110184)
Supplement: Supplementary file 1 [file bioengineering-08-00184-s001.zip › bioengineering-1425435-supplementary.pdf]

Heat-Inactivation of Fetal and Newborn Sera Did Not Impair the Expansion or Scaffold Engineering Potentials of Fibroblasts

Felix-Antoine Pellerin, Christophe Caneparo, Ève Pellerin, Stéphane Chabaud, Martin Pelletier and Stéphane Bolduc

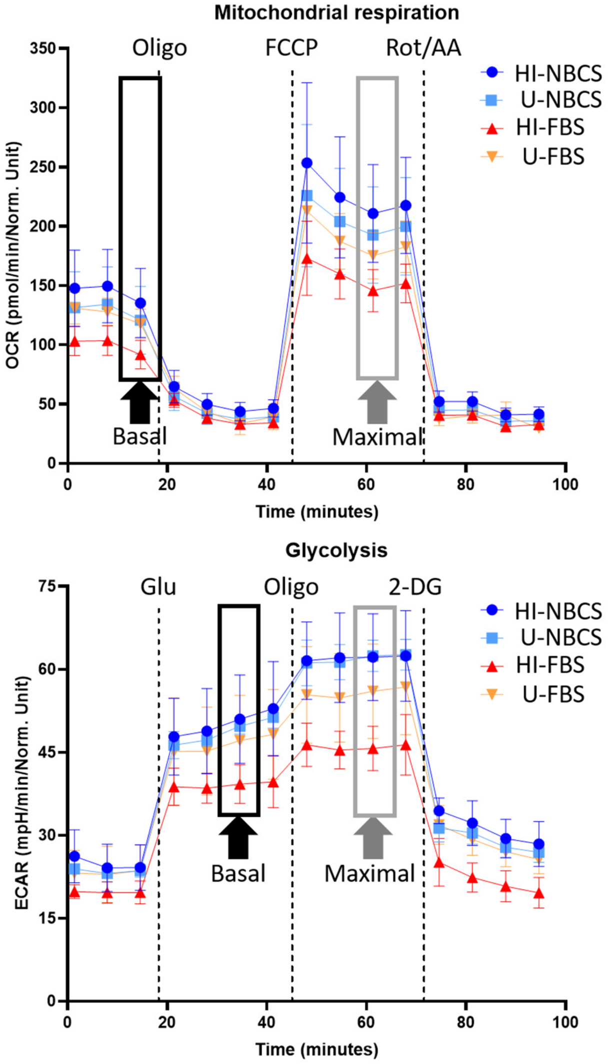

Figure S1. Example of test profiles (here DF1). Products injected were indicated as (Oligo, FCCP, and Rot/AA for mitochondrial respiration and Glu, Oligo, and 2-DG for glycolysis. HI- is for heat-inactivated, U- for untreated, FBS for fetal bovine serum, and NBCS for newborn calf serum. Measures between the start of the experiment and the injection of Oligo correspond to the basal respiration, and measures between FCCP and Rot/AA correspond to the maximal respiration. The measurement used in analyses for basal mitochondrial respiration determination corresponds to the last value before Oligo injection (black arrow). The measurement used in analyses for maximal mitochondrial respiration determination corresponds to the third value after FCCP injection (grey arrow). Measures between the injection of Glu and the injection of Oligo correspond to the basal glycolysis, and measures between Oligo and 2-DG correspond to the maximal glycolysis. The measurement used in analyses for basal glycolysis determination corresponds to the third value after Glu injection (black arrow). The measurement used in analyses for maximal glycolysis determination corresponds to the third value after Oligo injection (grey arrow).

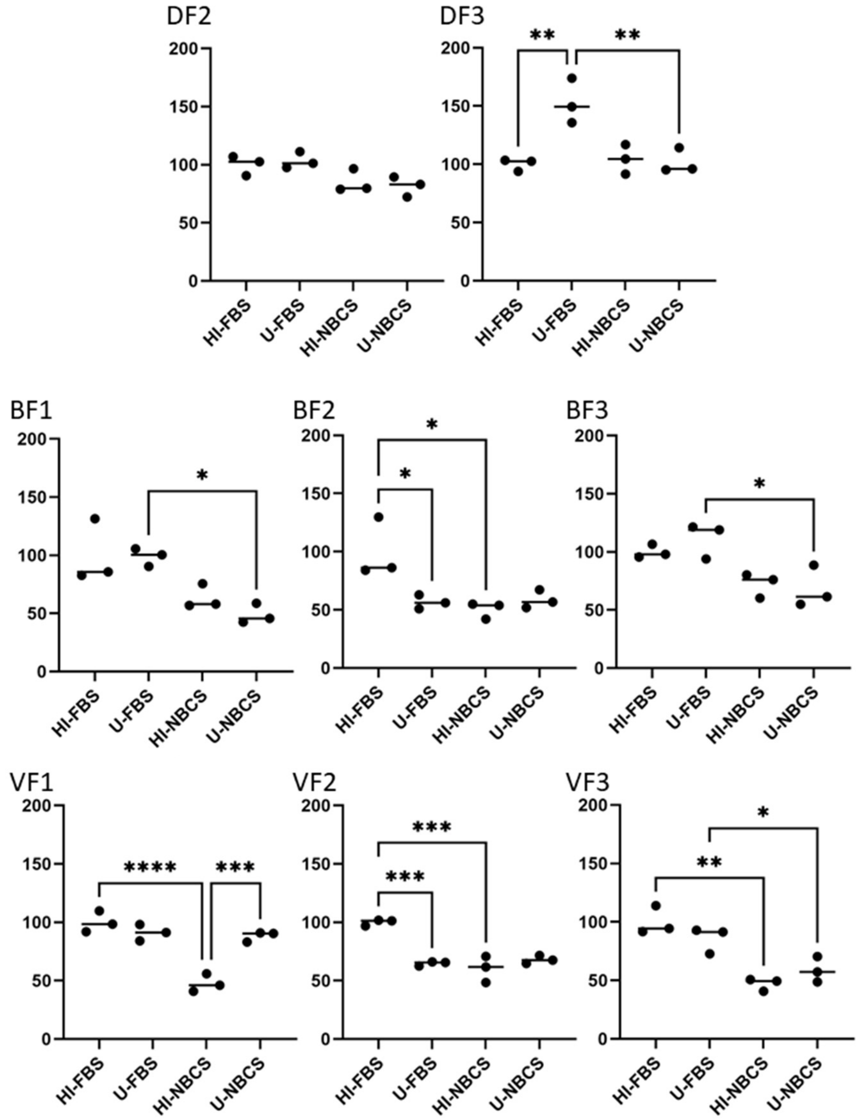

Figure S2. Adhesion of fibroblast cell populations on plastic Petri dish. DF1, DF2, and DF3 are fibroblast populations derived from skin dermis, BF1, BF2, and BF3 are fibroblast populations derived from bladder lamina propria, and VF1, VF2, and VF3 are fibroblast populations derived from vaginal stromae. HI- is for heat-inactivated, U- for untreated, FBS for fetal bovine serum, and NBCS for newborn calf serum. Each dot represents a cell count 24h after seeding normalized to the cell count obtained in HI-FBS. The line is the mean of the measures. Asterisks (*) illustrate significant differences. One asterisk for p-value between 0.05 and 0.01, 2 asterisks for p-value between 0.01 and 0.005, 3 asterisks for p-value 0.005 to 0.001, 4 asterisks for p-value inferior to 0.001.

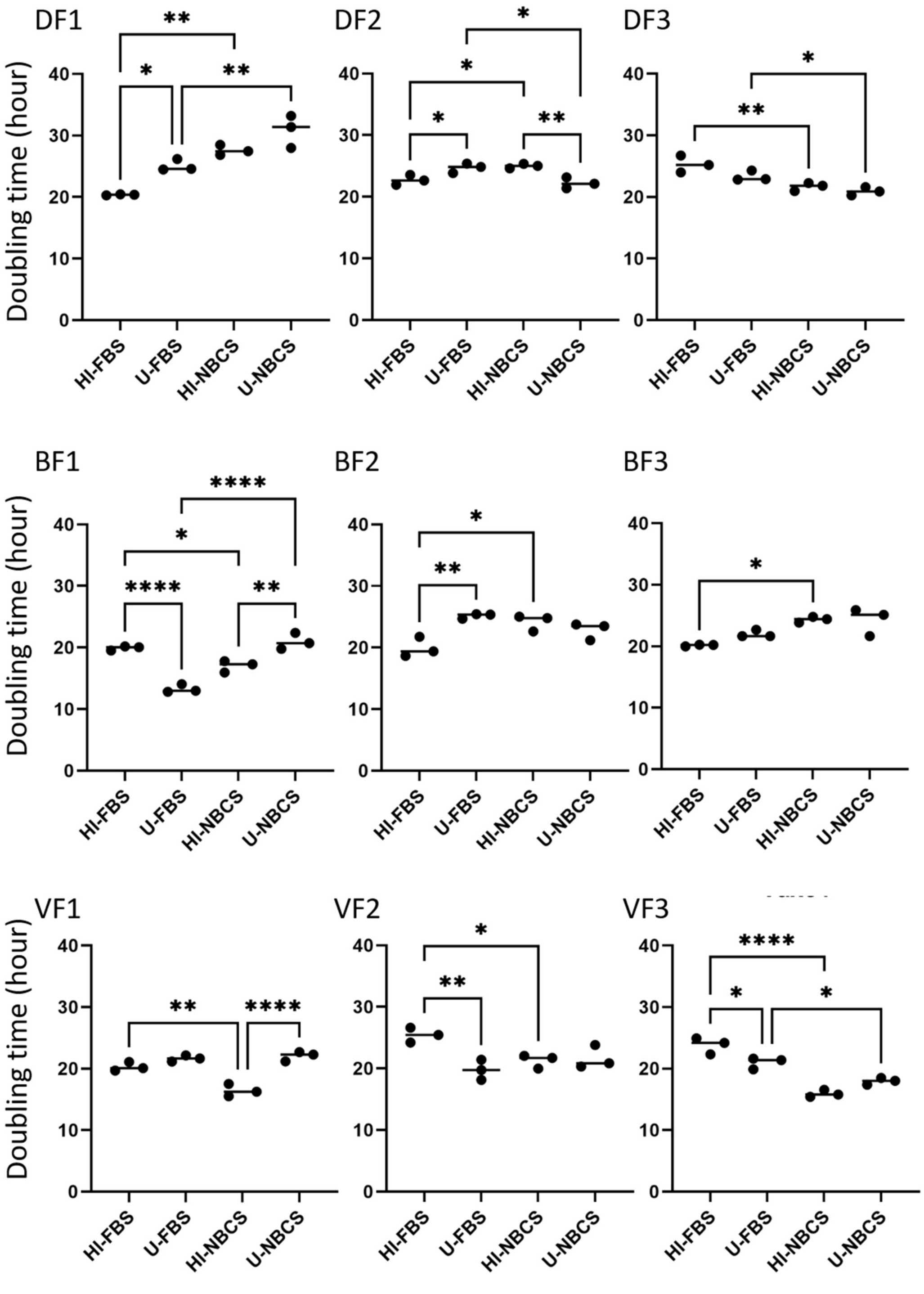

Figure S3. Proliferation of fibroblast cell populations on three days experiment. DF1, DF2, and DF3 are fibroblast populations derived from skin dermis, BF1, BF2, and BF3 are fibroblast populations derived from bladder lamina propria, and VF1, VF2, and VF3 are fibroblast populations derived from vaginal stromae. HI- is for heat-inactivated, U- for untreated, FBS for fetal bovine serum, and NBCS for newborn calf serum. Each dot represents the doubling time of the cell population calculated from a growth curve established during three days. The line is the mean of the measure. Asterisks (*) illustrate significant differences. One asterisk for p-value between 0.05 and 0.01, 2 asterisks for p-value between 0.01 and 0.005, 3 asterisks for p-value 0.005 to 0.001, 4 asterisks for p-value inferior to 0.001.

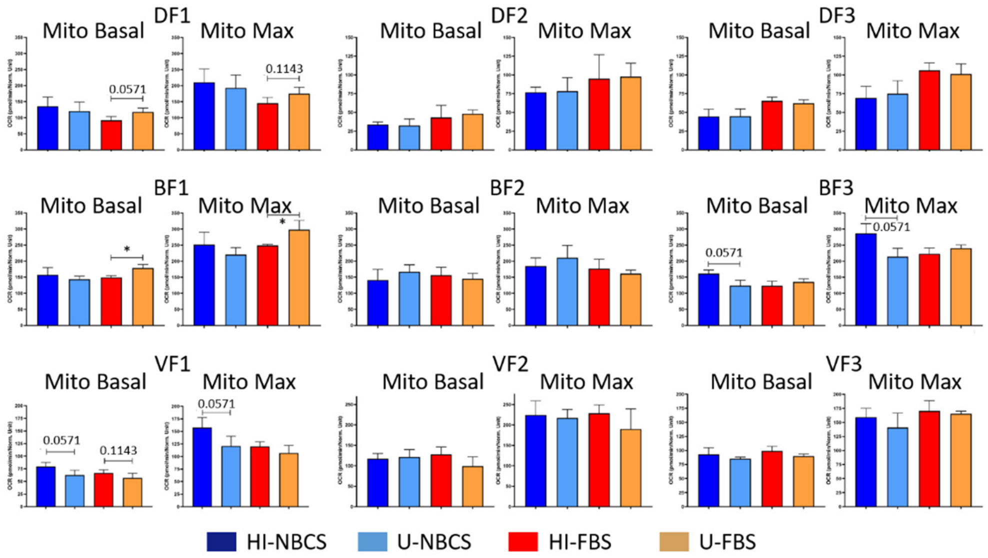

Figure S4. Mitochondrial activity of fibroblast cell populations. DF1, DF2, and DF3 are fibroblast populations derived from skin dermis, BF1, BF2, and BF3 are fibroblast populations derived from bladder lamina propria, and VF1, VF2, and VF3 are fibroblast populations derived from vaginal stromae. HI- is for heat-inactivated, U- for untreated, FBS for fetal bovine serum, and NBCS for newborn calf serum. The bars represent the mean +/- standard deviation of oxygen consumption rate (OCR) measured by a real-time extracellular flux analyzer. Basal mitochondrial activity and maximal mitochondrial activity were measured. Significant differences are illustrated by an asterisk (*) for a p-value inferior to 0.05. When a trend exists (but not a significant difference), the p-value is noted.

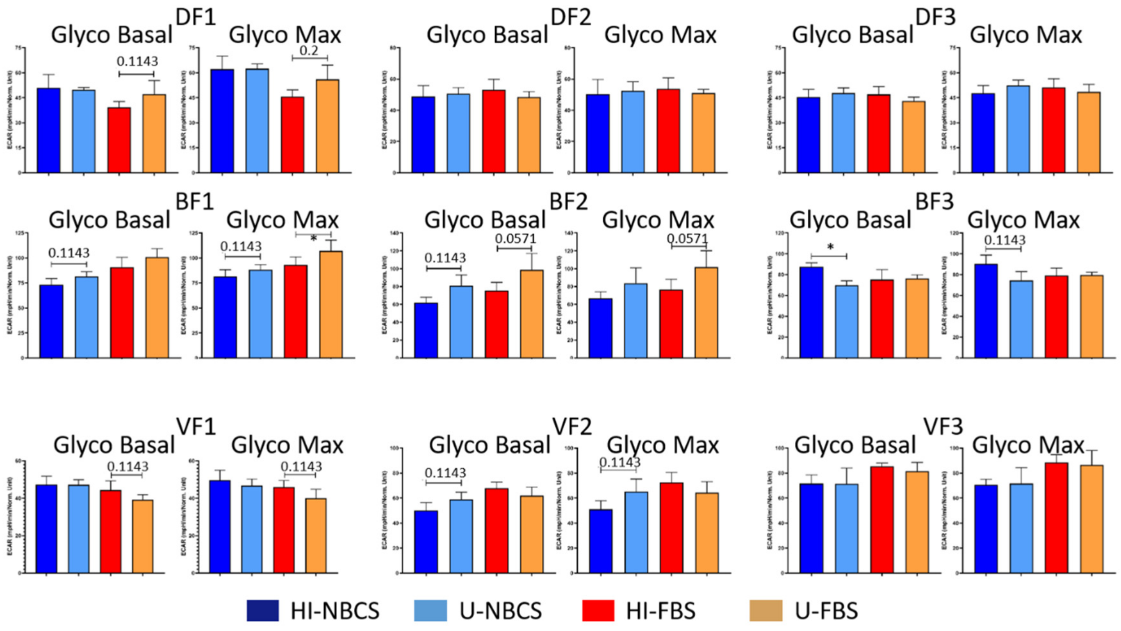

Figure S5. Glycolytic activity of fibroblast cell populations. DF1, DF2, and DF3 are fibroblast populations derived from skin dermis, BF1, BF2, and BF3 are fibroblast populations derived from bladder lamina propria, and VF1, VF2, and VF3 are fibro-blast populations derived from vaginal stromae. HI- is for heat-inactivated, U- for untreated, FBS for fetal bovine serum, and NBCS for newborn calf serum. The bars represent the mean +/- standard deviation of extracellular acidification rate (ECAR) measured by a real-time extracellular flux analyzer. Basal glycolytic activity and maximal glycolytic activity were measured. Significant differences are illustrated by an asterisk (*) for a p-value inferior to 0.05. When a trend exists (but not a significant difference), the p-value is noted.

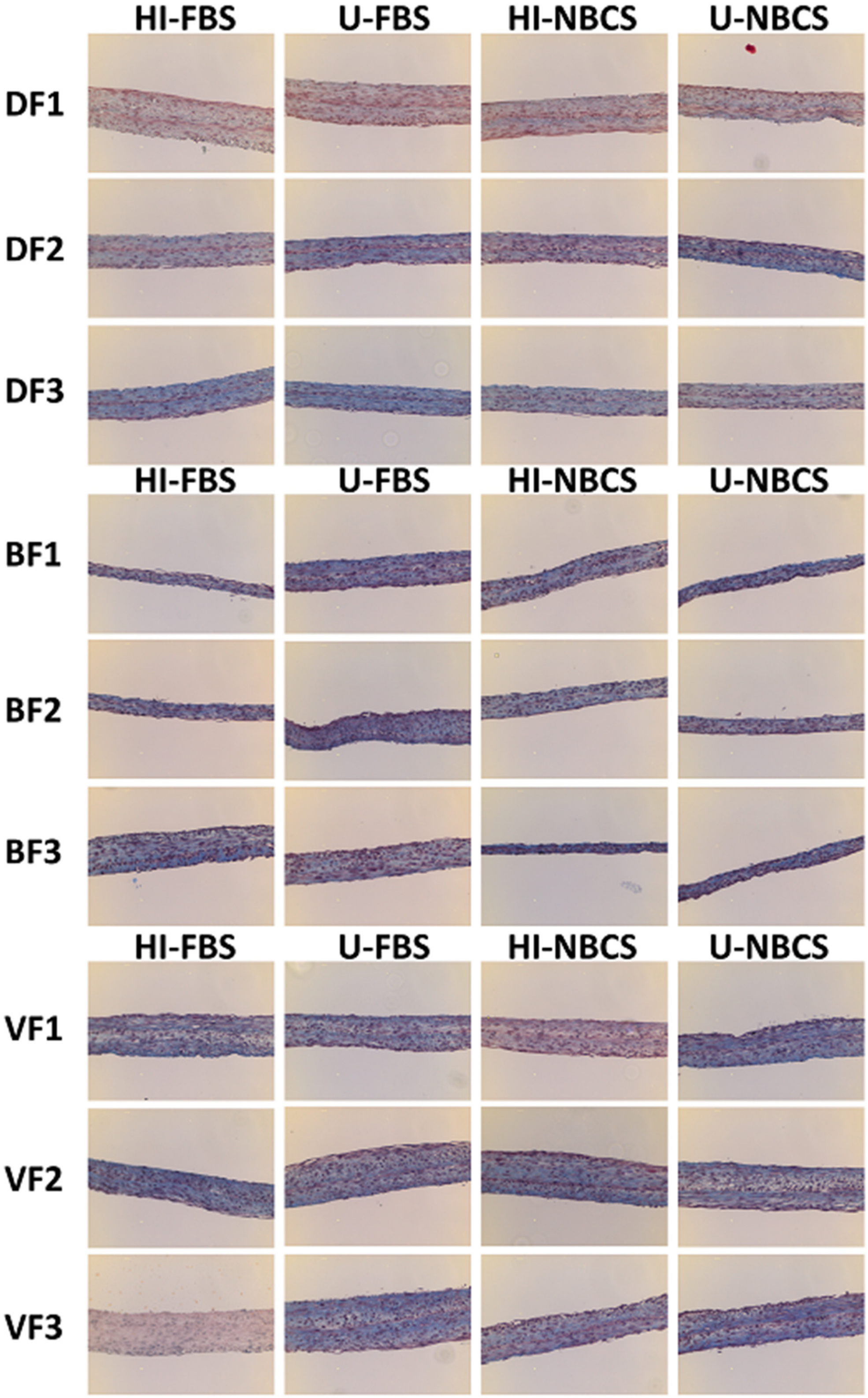

Figure S6. Histological aspect of reconstructed stromae stained with Masson's Trichrome protocol. DF1, DF2, and DF3 are fibroblast populations derived from skin dermis, BF1, BF2, and BF3 are fibroblast populations derived from bladder lamina, propria, and VF1, VF2, and VF3 are fibroblast populations derived from vaginal stromae. HI- is for heat-inactivated, U- for untreated, FBS for fetal bovine serum, and NBCS for newborn calf serum.

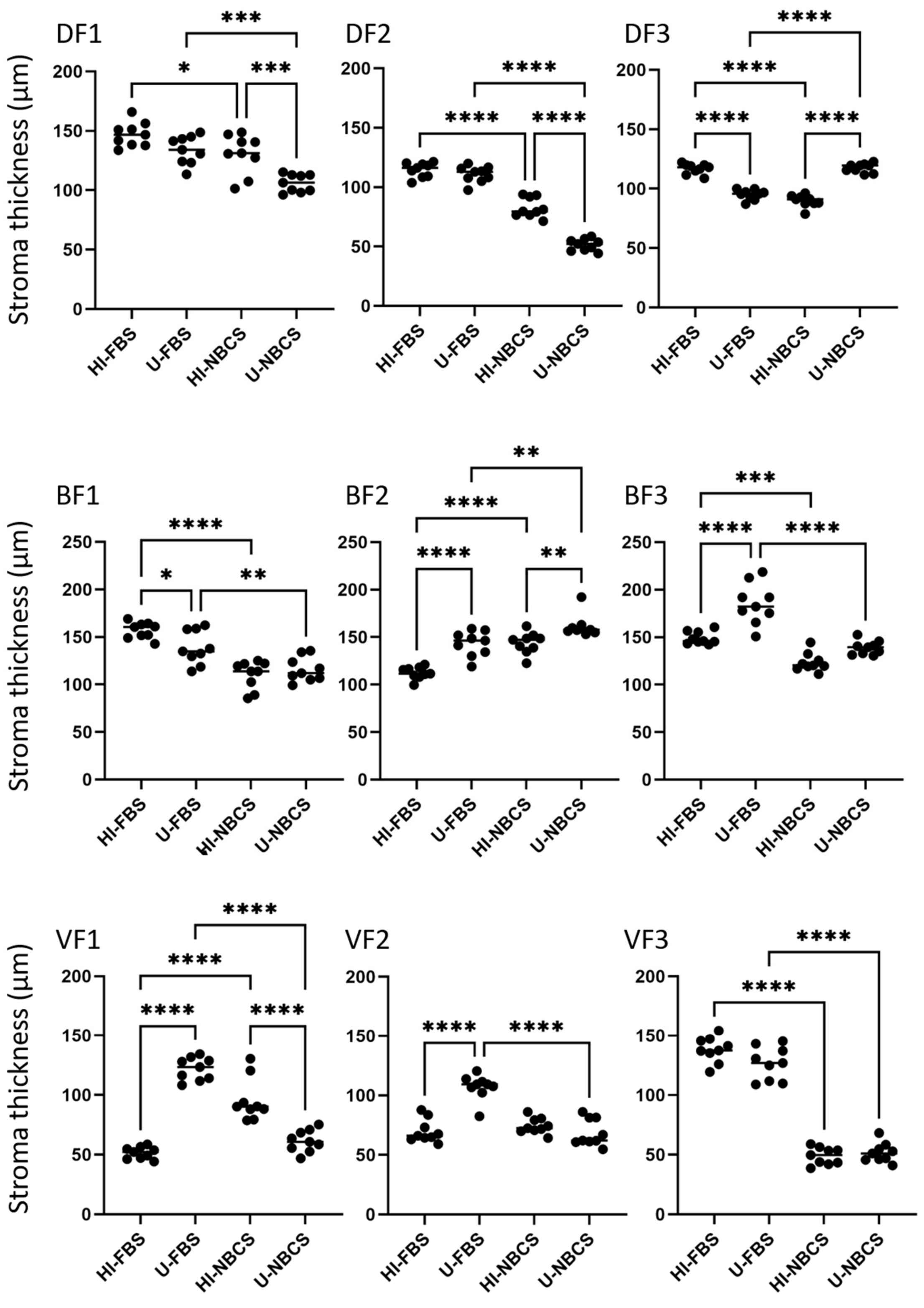

Figure S7. Stroma thickness of reconstructed stromae. DF1, DF2, and DF3 are fibroblast populations derived from skin dermis, BF1, BF2, and BF3 are fibroblast populations derived from bladder lamina propria, and VF1, VF2, and VF3 are fibroblast populations derived from vaginal stromae. HI- is for heat-inactivated, U- for untreated, FBS for fetal bovine serum, and NBCS for newborn calf serum. Each dot represents a measurement on a photograph of the tissue slices stained with the Masson's Trichrome protocol. The line is the mean of the measure. Asterisks (*) illustrate significant differences. One asterisk for p-value between 0.05 and 0.01, 2 asterisks for p-value between 0.01 and 0.005, 3 asterisks for p-value 0.005 to 0.001, 4 asterisks for p-value inferior to 0.001.

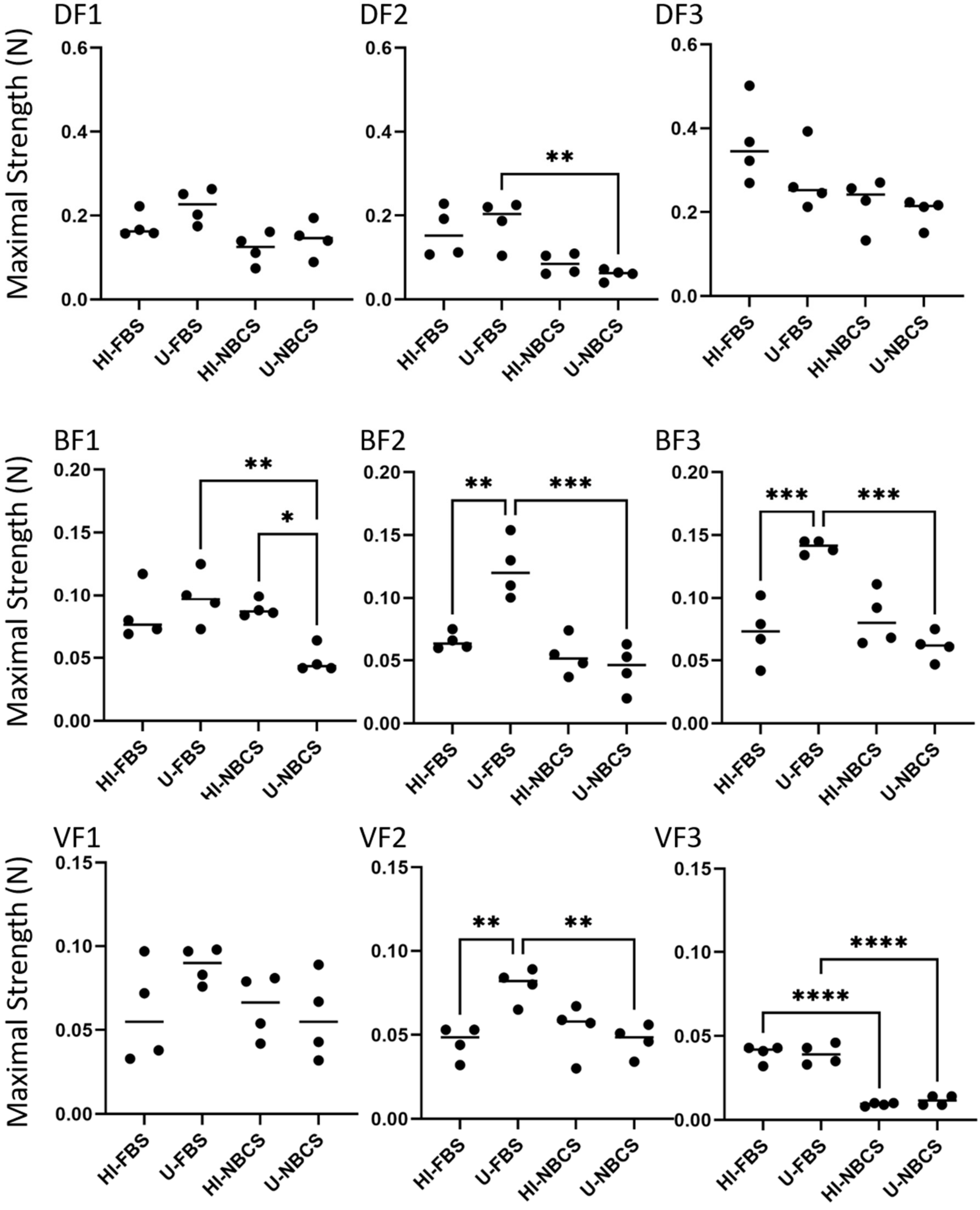

Figure S8. Maximal strength of reconstructed stromae. DF1, DF2, and DF3 are fibroblast populations derived from skin dermis, BF1, BF2, and BF3 are fibroblast populations derived from bladder lamina propria, and VF1, VF2, and VF3 are fibroblast populations derived from vaginal stromae. HI- is for heat-inactivated, U- for untreated, FBS for fetal bovine serum, and NBCS for newborn calf serum. Each dot represents a measurement of the maximal strength in Newton (N) using a uniaxial tensile test. The line is the mean of the measure. Asterisks (*) illustrate significant differences. One asterisk for p-value between 0.05 and 0.01, 2 asterisks for p-value be-tween 0.01 and 0.005, 3 asterisks for p-value 0.005 to 0.001, 4 asterisks for p-value inferior to 0.001.

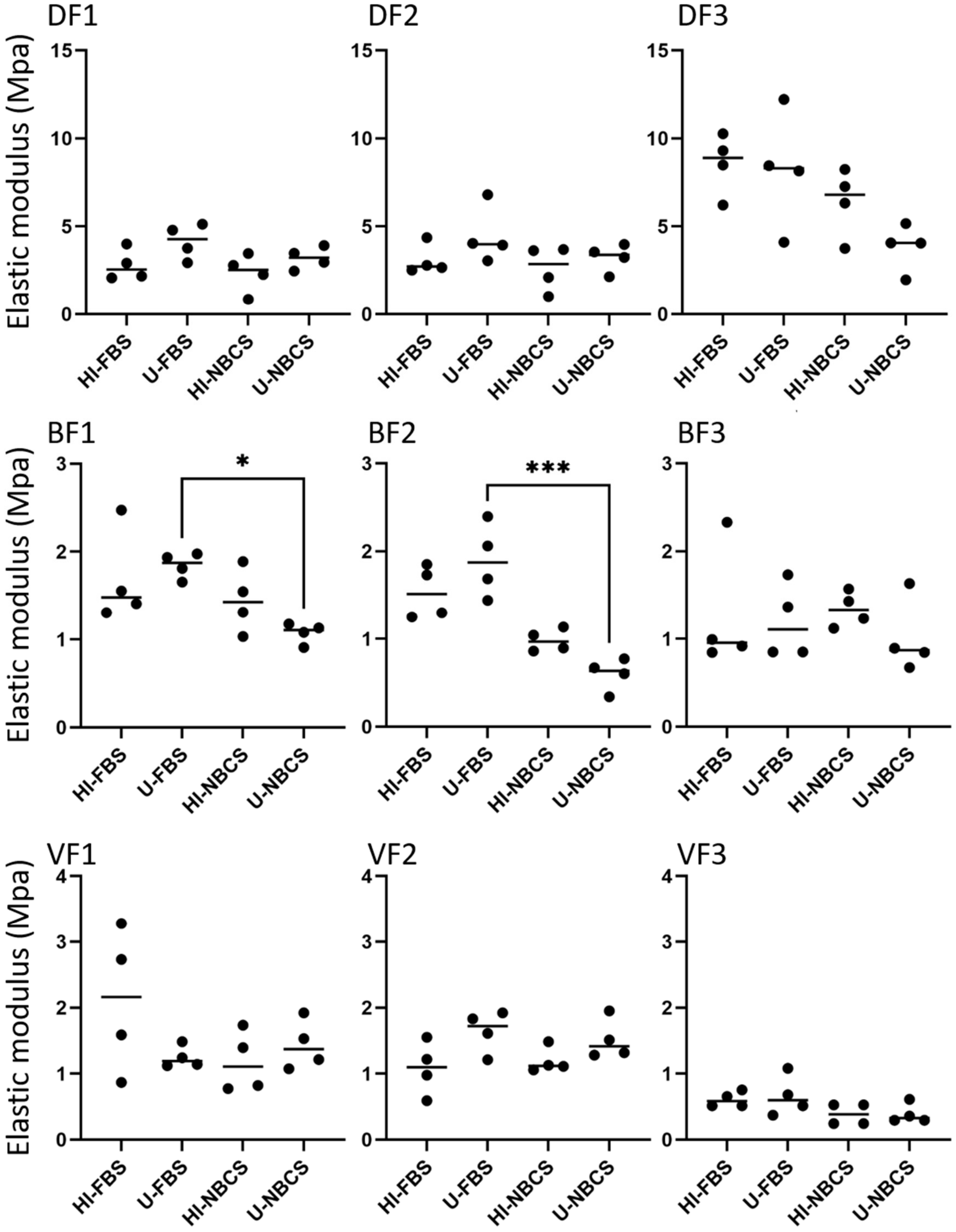

Figure S9. Elastic modulus of reconstructed stromae. DF1, DF2, and DF3 are fibroblast populations derived from skin dermis, BF1, BF2, and BF3 are fibroblast populations derived from bladder lamina propria, and VF1, VF2, and VF3 are fibroblast populations derived from vaginal stromae. HI- is for heat-inactivated, U- for untreated, FBS for fetal bovine serum, and NBCS for newborn calf serum. Each dot represents a measurement of the elastic modulus in MegaPascal (MPa) using a uniaxial tensile test. The elastic modulus is in inverse relation to elasticity. The line is the mean of the measure Asterisks (*) illustrate significant differences. One asterisk for p-value between 0.05 and 0.01, 2 asterisks for p-value be-tween 0.01 and 0.005, 3 asterisks for p-value 0.005 to 0.001, 4 asterisks for p-value inferior to 0.001.

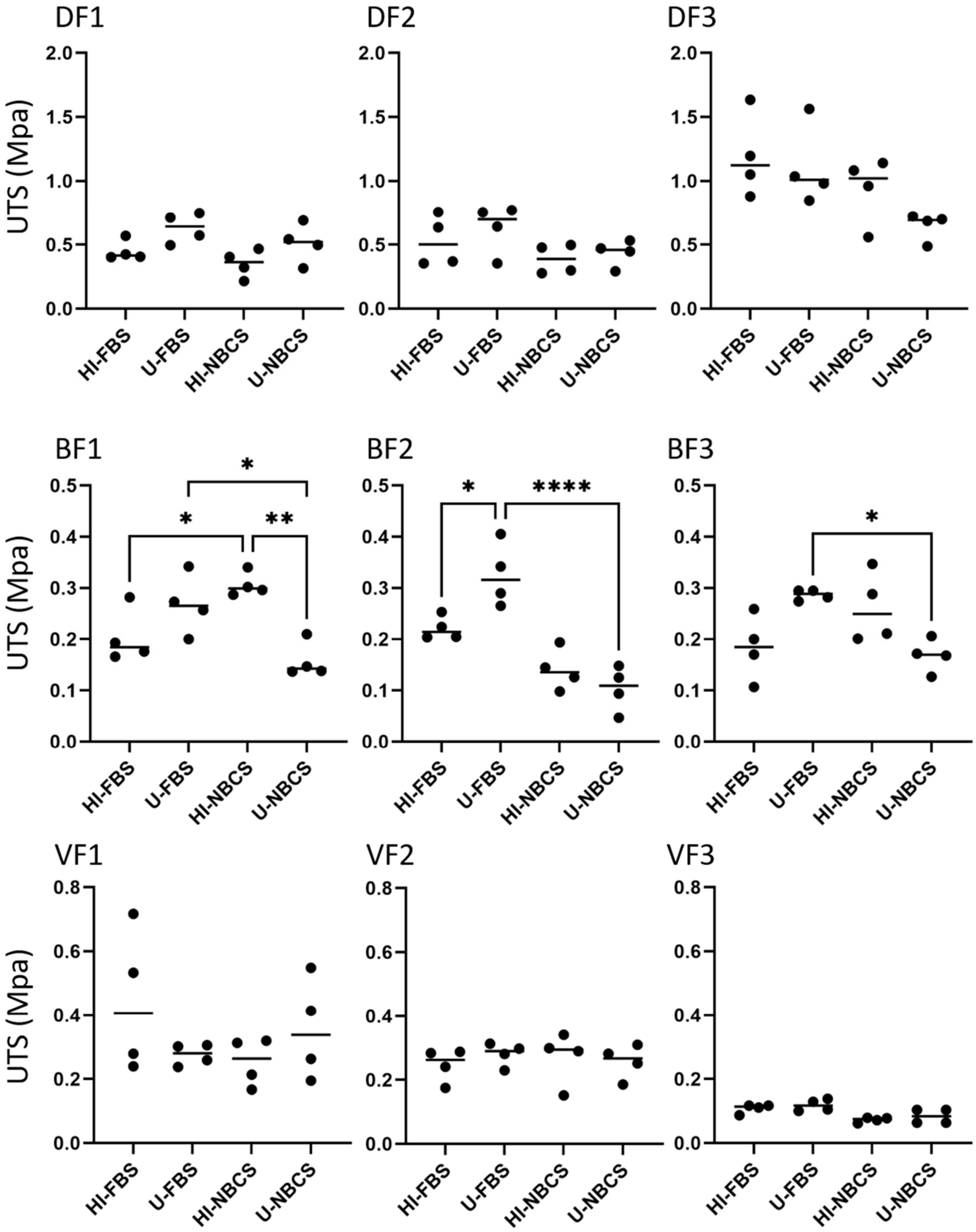

Figure S10. Ultimate tensile strength of reconstructed stromae. DF1, DF2 and DF3 are fibroblast populations derived from skin dermis, BF1, BF2, and BF3 are fibroblast populations derived from bladder lamina propria, and VF1, VF2, and VF3 are fibroblast populations derived from vaginal stromae. HI- is for heat-inactivated, U- for untreated, FBS for fetal bovine serum, and NBCS for newborn calf serum. Each dot represents a measurement of the UTS in MegaPascal (MPa) using a uniaxial tensile test. The line is the mean of the measure. Asterisks (*) illustrate significant differences. One asterisk for p-value between 0.05 and 0.01, 2 asterisks for p-value be-tween 0.01 and 0.005, 3 asterisks for p-value 0.005 to 0.001, 4 asterisks for p-value inferior to 0.001.

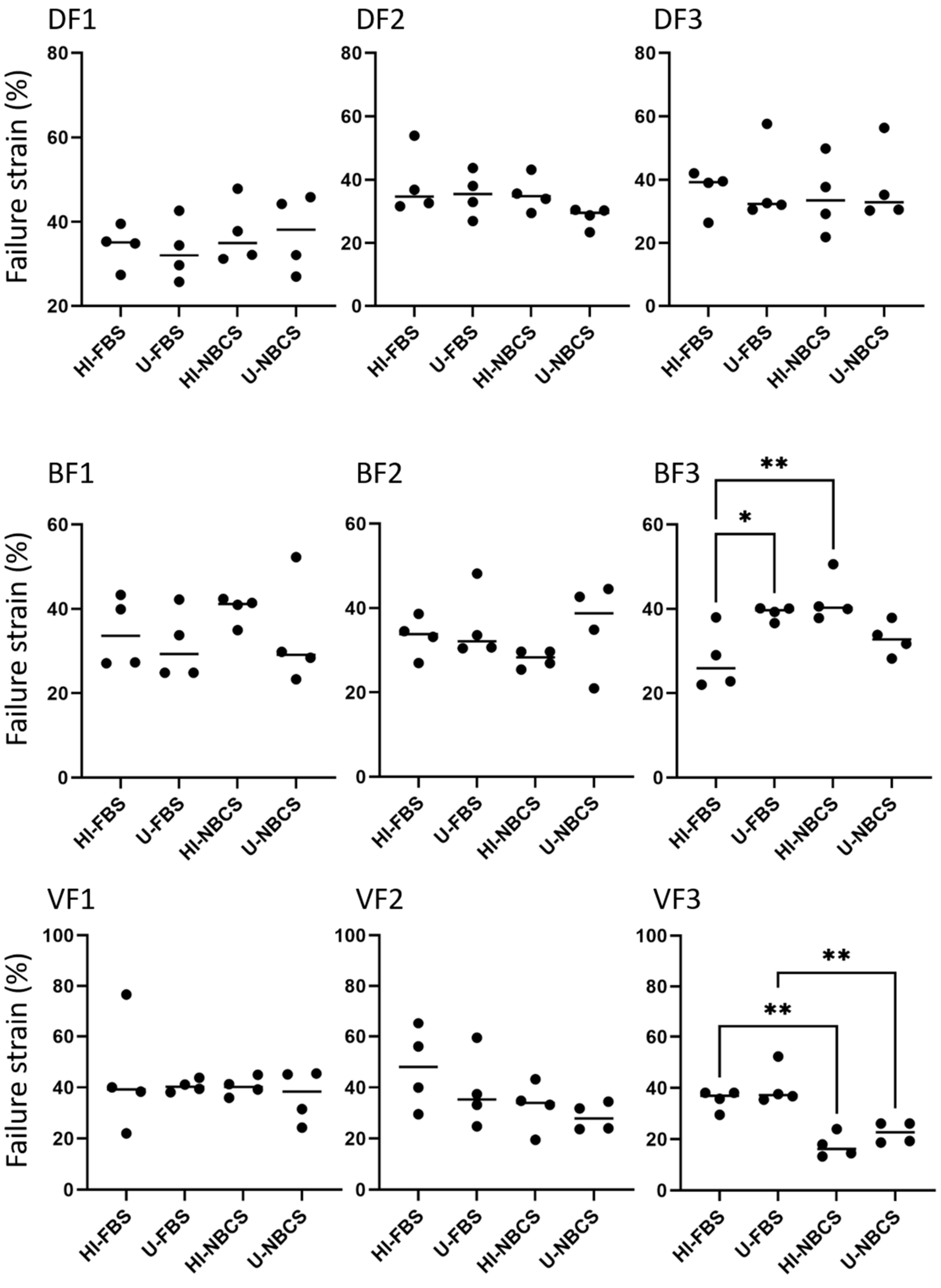

Figure S11. Failure strain of reconstructed stromae. DF1, DF2, and DF3 are fibroblast populations derived from skin dermis, BF1, BF2, and BF3 are fibroblast populations derived from bladder lamina propria, and VF1, VF2, and VF3 are fibroblast populations derived from vaginal stromae. HI- is for heat-inactivated, U- for untreated, FBS for fetal bovine serum, and NBCS for newborn calf serum. Each dot represents a measurement of the failure strain in the percentage of the tissue length (%) using a uniaxial tensile test. The line is the mean of the measure. Asterisks (*) illustrate significant differences. One asterisk for p-value between 0.05 and 0.01, 2 asterisks for p-value be-tween 0.01 and 0.005, 3 asterisks for p-value 0.005 to 0.001, 4 asterisks for p-value inferior to 0.001.
